# *In Vitro* Fertilization/Intracytoplasmic Sperm Injection with Autologous Oocytes in Healthy Women of Advanced Maternal Age: A Comparative Study Investigating Obstetric and Perinatal Outcomes Through Single Versus Double Embryo Transfer

**DOI:** 10.1089/whr.2023.0178

**Published:** 2024-06-27

**Authors:** Ellen-Elena Reinolds, Panagiotis Tsiartas, Nermin Hadziosmanovic, Kenny A. Rodriguez-Wallberg

**Affiliations:** ^1^ Division of Gynecology and Reproduction, Department of Reproductive Medicine, Karolinska University Hospital, Stockholm, Sweden.; ^2^ Department of Obstetrics and Gynecology, Södertälje Hospital, Stockholm, Sweden.; ^3^ Nordic IVF Solna, Eugin Group, Solna, Sweden.; ^4^ Division of Obstetrics and Gynecology, Department of Clinical Science, Intervention and Technology (CLINTEC), Karolinska Institute, Stockholm, Sweden.; ^5^ Clinical Research Center, Uppsala University, Uppsala, Sweden.; ^6^ Laboratory of Translational Fertility Preservation, Department of Oncology-Pathology, Karolinska Institute, Stockholm, Sweden.

**Keywords:** advanced maternal age, DET, *in vitro* fertilization, obstetric outcome, perinatal outcome, SET

## Abstract

**Introduction:**

The aim of this study was to assess whether the choice between double embryo transfer (DET) and single embryo transfer (SET) in healthy women of advanced maternal age (AMA) was associated with an increased risk of adverse outcomes.

**Materials and Methods:**

Healthy women aged 39–40 years who achieved live birth after *in vitro* fertilization (IVF)/intracytoplasmic sperm injection (ICSI) treatment between 2009 and 2020 at Karolinska University Hospital, Stockholm in Sweden, were included in this prospective, single-center cohort study.

**Results:**

A total of 310 women, who underwent IVF/ICSI treatments and achieved live births, were included in our analysis. Within this cohort, 78% of the women received SET, while 22% received DET. Nulliparity was common in both the SET (62.7%) and DET (85.3%) groups. Fresh embryo transfers were more prevalent in the DET group (91.2%) than in the SET group (31.1%). The rate of pregnancy-induced hypertension was higher in the SET group (8.3%) compared to the DET group (1.5%, *p* = 0.048). Furthermore, the DET group had a significantly higher rate of twin pregnancies (13.2%) compared to the SET group (0.4%). No statistically significant differences were observed in composite obstetric and perinatal complications between the SET and DET groups across all model estimates following different adjustments.

Clinical Trial Registration number: ClinicalTrials.gov NTC04602962.

**Conclusions:**

While DET was more common in nulliparous women and associated with a higher rate of twin pregnancies, our analysis did not reveal significant differences in adverse outcomes between the SET and DET groups after comprehensive adjustments. Our study suggests that in the absence of co-morbidities, meticulous patient selection coupled with comprehensive maternal care can potentially mitigate potential DET-associated risks in women of AMA.

## Introduction

In recent decades, Western societies have witnessed a significant increase in the number of women choosing to postpone childbirth beyond the age of 35.^1^ This trend can be attributed to several factors, including women's career aspirations, the pursuit of parity in the workforce, and the attainment of financial independence.^1^ This shift in family planning has brought about a corresponding rise in pregnancy complications and unfavorable outcomes.^2–4^ Studies have consistently linked nulliparity, the state of never having given birth, with an elevated risk of experiencing adverse obstetric and perinatal events, including conditions as preeclampsia, gestational diabetes, placental abruption, preterm birth, and the need for medical interventions during delivery.^5^ Conversely, in lower-income countries, advanced maternal age (AMA) does not necessarily deter childbearing, often resulting in high-order multiparous pregnancies due to cultural acceptance of larger family sizes and reduced use of family planning methods.^6^

The term “advanced maternal age” is commonly defined in studies as being 35 years or older at the time of delivery.^7^ For this study, AMA was defined as maternal age older than 35 years. According to previous studies, AMA pregnancies are at an increased risk of many obstetric and perinatal complications, including hypertensive disorders, gestational diabetes, placentation abnormalities, interventional deliveries, low birth weight, and higher maternal mortality.^1^ The combination of AMA and nulliparity has been associated with an elevated risk of perinatal complications.^7–10^ One study has demonstrated that obstetric complications in pregnancies among women of AMA who underwent *in vitro* fertilization (IVF) are higher compared to those in younger women.^11^

According to the European IVF-Monitoring Consortium, Europe witnessed a distribution of 34.9% single embryo transfer (SET) and 54.5% double embryo transfer (DET) during IVF treatments in 2014. This approach led to 82.5% singleton and 17% twin pregnancies in the same year.^12^ Multiple pregnancies are associated with increased maternal and perinatal morbidity, such as, preterm birth, low Apgar scores, and low birthweight.^13^ Notably, prior research has suggested that the perinatal outcomes of women of AMA with twin pregnancies resulting from IVF are similar to those of naturally conceived twin pregnancies within the same age group.^14^ Additionally, DET has been linked to a higher risk for adverse obstetric and perinatal outcomes, even when a singleton birth is achieved.^15^

In Sweden, and several other countries, guidelines recommend SET for younger women with favorable prognoses undergoing IVF/intracytoplasmic sperm injection (ICSI). However, the practice is more lenient for women of AMA, particularly if they are nulliparous, as tax-funded health care coverage for these treatments is no longer provided for women above 40 years of age. In Sweden, IVF/ICSI treatments are extended through the tax-funded health care system to couples encountering primary infertility, with eligibility for women below the age of 40 years. The study aimed to assess obstetric and perinatal outcomes in women of AMA without antenatal co-morbidities who achieved live births through IVF/ICSI treatments. Specifically, the study focused on comparing the outcomes between DET and SET within this cohort.

## Materials and Methods

This study prospectively focused on a specific cohort of women aged 39–40 years who underwent IVF/ICSI treatment at the Reproductive Medicine Unit of Karolinska University Hospital, Stockholm, Sweden, between January 2009 and January 2020, and subsequently achieved live births. The rationale behind this age subset is rooted in the intricacies of the Swedish health care system, where IVF/ICSI treatments are accessible through the tax-funded system for couples facing primary infertility, with eligibility extending up to the age of 40 years. Consequently, we aimed to examine the outcomes of IVF/ICSI treatments in the unique context of women between 39 and 40 years, as they represent the final age range eligible for these treatments within the tax-funded health care framework. For all IVF/ICSI procedures involving heterosexual couples, autologous gametes were utilized. In instances of same-sex couples and single women, the recipient's own oocytes were paired with donated sperm.

The donated sperm was obtained from anonymous donors aged between 23 and 45 years, who underwent meticulous medical and psychosocial evaluations. Before implementation in treatments, the sperm's quality underwent through validation processes.

Information pertaining to IVF/ICSI treatments was extracted from the electronic database “Linnéfiler” (Fertisoft AB, Uppsala, Sweden). Details regarding prior medical history and the causes of infertility were obtained from the medical records system “Take care” (CompuGroup Medical Sweden AB, Stockholm, Sweden). Obstetric and perinatal outcome data were collected by employing ICD-10 codes from the regional registry “Obstetrix” (Cerner Sweden AB, Stockholm, Sweden). Utilizing the distinct personal identity number assigned to Swedish citizens enabled the linkage between databases, facilitating the retrieval of comprehensive obstetric and perinatal outcome data.

The compiled dataset encompassed variables such as the woman's age at the time of IVF/ICSI treatment, body mass index (BMI), smoking habits, preexisting hypertension and diabetes, previous parity, serum anti-Müllerian hormone concentrations, mean antral follicle count, the specific type of gonadotropin releasing hormone protocol (agonist or antagonist), total follicle stimulating hormone dosage, the count of retrieved oocytes during oocyte retrieval, the nature of IVF treatment (conventional IVF, ICSI, or a combination of both), the type of embryo transfer (fresh or frozen/thawed), the number of embryo(s) transferred, obstetric outcomes (including pregnancy-induced hypertension, preeclampsia, gestational diabetes, placental abruption, placenta previa, venous thromboembolism, preterm prelabor rupture of membranes, chorioamnionitis, fetal hypoxia, mode of delivery, postpartum hemorrhage, and intrapartum infection/sepsis), as well as perinatal outcomes data.

The duration of gestation was computed using the date of embryo transfer as the reference point.

### Statistical analyses

To compare the groups of single and DET, we employed the chi square test and Fisher's exact test for categorical variables, and the Wilcoxon test for continuous variables. Continuous data are presented as median ± interquartile range (IQR) (minimum, maximum), while categorical data are presented as counts (*n*) and percentages (%). For this study, we have chosen to present obstetric and perinatal outcomes as composite variables to increase the statistical power of our observations. For the analysis of these composite outcomes, we utilized logistic regression, and the resulting outcomes are reported as odds ratios (OR) along with their corresponding 95% confidence intervals (CI) and associated *p*-values. The SET group was used as the reference for comparative purposes. The outcomes were stratified into different model estimates, each accounting for various adjustments, including maternal age, number of previous children (nulliparous/multiparous), type of performed embryo transfer (fresh/frozen), and type of pregnancy (singleton/twin).

A two-sided *p*-value of <0.05 was considered statistically significant. All statistical analyses were performed using SAS version 9.4 (SAS Institute, Inc., North Carolina, USA).

### Institutional Review Board approval statement

This study was performed in line with the principles of the Declaration of Helsinki. Approval was granted by the Regional Ethics Committee of Stockholm (Dnr. 2011/1758-31/2).

## Results

**FIG. 1.**
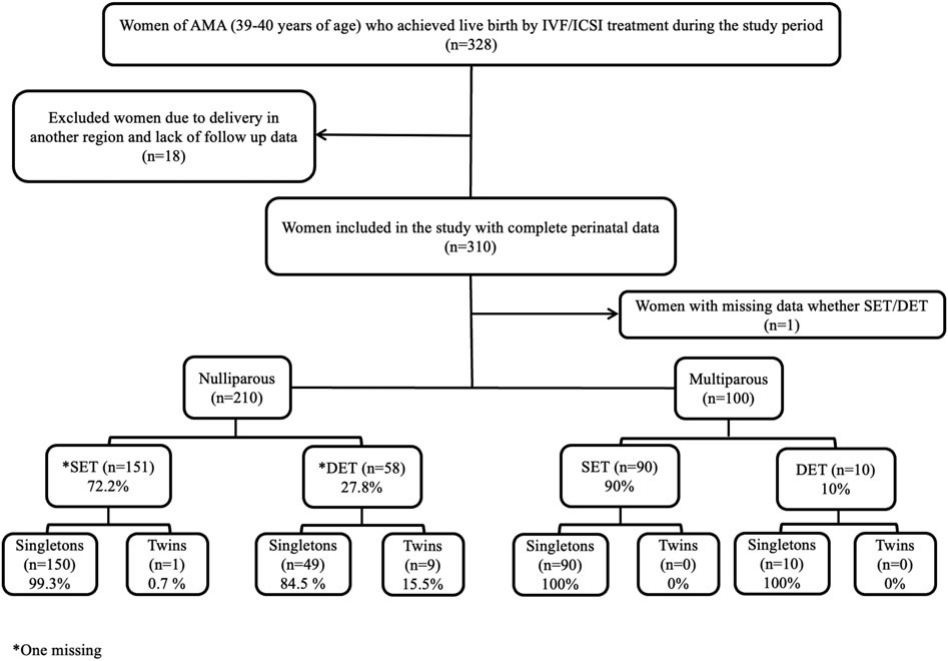
Flowchart of the study.

A total of 328 women who underwent IVF/ICSI treatment and were aged 39–40 years, achieving live births during the study period, were initially identified. Among these, 18 women who delivered in another county were lost to follow-up and subsequently excluded from the analyses due to unavailability of detailed obstetrical data. Consequently, a total of 310 women had complete perinatal data and were included in the analyses. Within the study cohort, there was one woman for whom data regarding whether she received SET or DET was missing (Fig. 1).

Table 1 summarizes descriptive statistics for baseline demographic, treatment, obstetric, and perinatal data, comparing women of AMA who received SET (*n* = 241, 78%) and DET (*n* = 68, 22%). Notable findings include a significant difference in maternal age between the two groups, with a median age of 39 years in both groups, but a narrower age range in the DET group (39–42 years) compared to the SET group (39–44 years). Among women of AMA, those undergoing SET and DET exhibited higher rates of nulliparity, with 62.7% and 85.3%, respectively. There were also significant differences in the type of performed embryo transfer, with more fresh transfers in the DET group (91.2%) compared to the SET group (31.1%).

**Table 1.** Descriptive Statistics of Demographic, Treatment, Obstetric, and Perinatal Data for Women Aged 39–40 Undergoing *In Vitro* Fertilization and Intracytoplasmic Sperm Injection Treatment with Single and Double Embryo Transfer

	SET (***n*** = 241)	DET (***n*** = 68)	***p***
Maternal age (years)	39 ± 1 (39, 44)	39 ± 0 (39, 42)	**0.002**
BMI (kg/m^2^)	24.6 ± 6 (18, 36)	24.3 ± 5 (19, 34)	0.97
Hypertension, *n* (%)	7 (2.9)	0 (0)	0.16
Diabetes, *n* (%)	1 (0.4)	0 (0)	0.59
Smoking, *n* (%)	0 (0)	3 (4.4)	**0.001**
AMH (μg/L)	1.9 ± 1.5 (0, 15)	1.2 ± 2.5 (0, 8)	**0.001**
Antral follicle count	2 ± 7 (1, 21)	7 ± 4.5 (3, 17)	**<0.0001**
Previous children, *n* (%)			**0.004**
Nulliparous	151 (62.7)	58 (85.3)	
Multiparous	90 (37.3)	10 (14.7)	
Stimulation protocol, *n* (%)			0.79
Agonist	54 (73)	44 (71)	
Antagonist	20 (27)	18 (29)	
Total FSH dose (IU)	3000 ± 2125 (150, 6188)	3894 ± 2325 (1125, 6300)	**0.006**
Total no. of oocytes retrieved at OPU	11 ± 8 (1, 46)	8 ± 5 (2, 20)	**0.0006**
Type of performed treatment, *n* (%)			0.010
IVF	130 (66.3)	31 (48.4)	
ICSI	62 (31.6)	33 (51.6)	
Combination IVF/ICSI	4 (2)	0 (0)	
Type of performed embryo transfer, *n* (%)			**<0.0001**
Frozen/thawed	166 (68.9)	6 (8.8)	
Fresh	75 (31.1)	62 (91.2)	
Pregnancy-induced hypertension, *n* (%)	20 (8.3)	1 (1.5)	**0.048**
Preeclampsia, *n* (%)	10 (4.1)	6 (8.8)	0.12
Gestational diabetes, *n* (%)	5 (2.1)	2 (2.9)	0.67
Placental abruption, *n* (%)	1 (0.4)	2 (2.9)	0.06
Placenta previa, *n* (%)	6 (2.5)	1 (1.5)	0.62
Venous thromboembolism, *n* (%)	1 (0.4)	2 (2.9)	0.06
PPROM, *n* (%)	4 (1.7)	2 (2.9)	0.49
Chorioamnionitis, *n* (%)	4 (1.7)	2 (2.9)	0.50
Fetal hypoxia, *n* (%)	26 (10.8)	11 (16.2)	0.23
Intrapartum infection/sepsis, *n* (%)	0 (0)	1 (1.5)	0.05
Postpartum hemorrhage, *n* (%)	41 (17)	13 (19.1)	0.69
Apgar score 1 minute ≤7, *n* (%)	27 (11.3)	10 (14.7)	0.44
Apgar score 5 minutes ≤7, *n* (%)	3 (1.3)	1 (1.5)	0.88
Birth weight child 1 < 2500 g, *n* (%)	7 (2.9)	6 (9.1)	**0.027**
Birth weight child 2 < 2500 g, *n* (%)	1 (100)	5 (44.1)	1.00
Mode of delivery, *n* (%)			0.92
Vaginal delivery	133 (55.4)	35 (52.2)	
Elective cesarean section	57 (23.8)	16 (23.9)	
Acute cesarean section	34 (14.2)	10 (14.9)	
Instrumental delivery	16 (6.7)	6 (9)	
Type of pregnancy, *n* (%)			**<0.0001**
Singleton	240 (99.6)	59 (86.8)	
Twin	1 (0.4)	9 (13.2)	
Gestational age at birth (weeks ± days)	40 ± 3 (25, 42)	39 ± 2 (31, 41)	**0.041**

Statistically significant *p* values are indicated in bold.

Analyses were performed with chi square test and Fisher's exact test for categorical variables and Wilcoxon test for continuous variables. Results are presented as median ± IQR (minimum, maximum) for continuous variables and as number (*n*) and percentage (%) for categorical variables.

AMH, anti-Müllerian hormone; BMI, body mass index; DET, double embryo transfer; FSH, follicle stimulating hormone; ICSI, intracytoplasmic sperm injection; IQR, interquartile range; IVF, *in vitro* fertilization; OPU, oocyte pick-up; PPROM, preterm prelabor rupture of membranes; SET, single embryo transfer.

The rate of pregnancy-induced hypertension was higher in the SET group compared with the DET group (8.3% vs. 1.5%, *p* = 0.048). Moreover, the DET group had a significantly higher rate of twin pregnancies (13.2%) compared to the SET group (0.4%). Gestational age at birth was slightly lower in the DET group (39 ± 2 weeks) compared to the SET group (40 ± 3 weeks).

Table 2 presents a comprehensive analysis of composite obstetric and perinatal outcomes. No statistically significant differences were found in composite pregnancy complications, including pregnancy-induced hypertension, preeclampsia, gestational diabetes, placental abruption, placenta previa, venous thromboembolism, and preterm prelabor rupture of membranes, between the SET and DET groups across all model estimates. Similarly, composite pregnancy and intrapartum complications, composite intrapartum complications, composite pregnancy, intrapartum, and postpartum complications, and composite postpartum and neonatal complications did not exhibit statistically significant differences between the two groups across different adjustments.

**Table 2.** Composite Obstetric and Perinatal Outcomes of *In Vitro* Fertilization and Intracytoplasmic Sperm Injection in Women Initiating Treatment at the Age of 39–40 Years: Single Embryo Transfer Versus Double Embryo Transfer

	Model estimate 1	Model estimate 2	Model estimate 3	Model estimate 4	Model estimate 5	Model estimate 6
	Unadjusted	Adjusted for maternal age	Adjusted for the number of previous children (nulliparous/multiparous)	Adjusted for the type of performed embryo transfer (fresh/frozen)	Adjusted for the type of pregnancy (singleton/twin)	Adjusted for maternal age, number of previous children (nulliparous/multiparous), type of performed embryo transfer (fresh/frozen), type of pregnancy (singleton/twin)
Composite pregnancy complications (pregnancy-induced hypertension, preeclampsia, gestational diabetes, placental abruption, placenta previa, venous thromboembolism, PPROM)	OR 1.11, 95% CI 0.54–2.26, *p* = 0.77	OR 1.15, 95% CI 0.56–2.36, *p* = 0.71	OR 1.10, 95% CI 0.53–2.28, *p* = 0.80	OR 1.43, 95% CI 0.61–3.36, *p* = 0.41	OR 1.08, 95% CI 0.51–2.29, *p* = 0.84	OR 1.37, 95% CI 0.57–3.31, *p* = 0.48
Composite pregnancy and intrapartum complications (pregnancy-induced hypertension, preeclampsia, gestational diabetes, placental abruption, placenta previa, venous thromboembolism, PPROM, chorioamnionitis, fetal hypoxia, intrapartum infection/sepsis)	OR 0.99, 95% CI 0.57–1.73, *p* = 0.98	OR 1.09, 95% CI 0.62–1.93, *p* = 0.76	OR 0.98, 95% CI 0.55–1.73, *p* = 0.94	OR 1.11, 95% CI 0.58–2.13, *p* = 0.75	OR 1.04, 95% CI 0.58–1.86, *p* = 0.91	OR 1.09, 95% CI 0.56–2.14, *p* = 0.79
Composite intrapartum complications (chorioamnionitis, fetal hypoxia, intrapartum infection/sepsis)	OR 1.13, 95% CI 0.62–2.04, *p* = 0.69	OR 1.25, 95% CI 0.68–2.30, *p* = 0.46	OR 1.09, 95% CI 0.59–1.99, *p* = 0.79	OR 0.99, 95% CI 0.50–1.96, *p* = 0.98	OR 1.12, 95% CI 0.60–2.09, *p* = 0.72	OR 0.92, 95% CI 0.46–1.87, *p* = 0.83
Composite pregnancy, intrapartum, and postpartum complications (pregnancy-induced hypertension, preeclampsia, gestational diabetes, placental abruption, placenta previa, venous thromboembolism, PPROM, chorioamnionitis, fetal hypoxia, intrapartum infection/sepsis, postpartum hemorrhage)	OR 1.15, 95% CI 0.64–2.09, *p* = 0.64	OR 1.25, 95% CI 0.68–2.29, *p* = 0.47	OR 1.12, 95% CI 0.61–2.05, *p* = 0.72	OR 0.99, 95% CI 0.50–1.97, *p* = 0.98	OR 1.15, 95% CI 0.61–2.14, *p* = 0.67	OR 0.94, 95% CI 0.47–1.91, *p* = 0.87
Composite postpartum and neonatal complications (postpartum hemorrhage, Apgar score 1 minute ≤7, Apgar score 5 minutes ≤7, birth weight child 1 < 2500 g, birth weight child 2 < 2500 g)	OR 1.15, 95% CI 0.64–2.04, *p* = 0.65	OR 1.23, 95% CI 0.68–2.21, *p* = 0.49	OR 1.15, 95% CI 0.64–2.08, *p* = 0.64	OR 1.21, 95% CI 0.62–2.38, *p* = 0.57	OR 0.88, 95% CI 0.47–1.66, *p* = 0.69	OR 0.93, 95% CI 0.45–1.90, *p* = 0.84
Composite pregnancy, intrapartum, postpartum, and neonatal complications (pregnancy-induced hypertension, preeclampsia, gestational diabetes, placental abruption, placenta previa, venous thromboembolism, PPROM, chorioamnionitis, fetal hypoxia, intrapartum infection/sepsis, postpartum hemorrhage, Apgar score 1 minute ≤7, Apgar score 5 minutes ≤7, birth weight child 1 < 2500 g, birth weight child 2 < 2500 g)	OR 0.97, 95% CI 0.57–1.67, *p* = 0.92	OR 1.04, 95% CI 0.60–1.80, *p* = 0.89	OR 0.95, 95% CI 0.55–1.65, *p* = 0.85	OR 1.06, 95% CI 0.56–1.98, *p* = 0.86	OR 0.83, 95% CI 0.47–1.48, *p* = 0.53	OR 0.89, 95% CI 0.46–1.70, *p* = 0.71

The statistical analyses were performed utilizing logistic regression. The resultant outcomes are presented as OR accompanied by their corresponding 95% CI and *p*-values. The SET is utilized as the reference group for comparative purposes.

CI, confidence intervals; OR, odds ratios; PPROM, Preterm Prelabor Rupture of Membranes; SET, single embryo transfer.

## Discussion

Changes in socioeconomic circumstances have led to a trend of delayed childbearing, which has been accompanied by an increase in adverse pregnancy outcomes.^2–4^ In this comprehensive study, we conducted a thorough comparison of composite obstetric and perinatal outcomes among women of AMA who underwent IVF/ICSI treatments, specifically examining those who received SET or DET with homologous oocytes at a single center in Sweden during the period from 2009 to 2020. However, we did not investigate the efficacy of either SET or DET in this population. To the best of our knowledge, this study represents the first effort to analyze and compare obstetric and perinatal outcomes in women of AMA undergoing IVF/ICSI treatment, considering the number of embryos transferred.

Among the cohort of women included in our analysis, SET was the chosen strategy in most cases, accounting for 78% of the total. This aligns with the prevailing national guidelines for IVF/ICSI treatments. It is noteworthy that women who successfully delivered following SET were predominantly nulliparous, constituting 63% of this group. Additionally, they more frequently received frozen-thawed embryos, resulting in most pregnancies after SET being singleton pregnancies, totaling 97%. Conversely, DET was performed in 22% of cases, and women in this group were more frequently nulliparous, comprising 85% of the DET cohort. Furthermore, fresh embryos were more commonly utilized for embryo transfer in DET cases, accounting for 91% of procedures. Consequently, women who received DET experienced twin pregnancies in ∼13% of cases.

However, despite the variations in embryo transfer strategies and pregnancy outcomes between SET and DET groups, our extensive analyses revealed no statistically significant differences in numerous obstetric and perinatal complications after adjustment for various parameters in this cohort of women of AMA.

In our study, the majority of women of AMA undergoing IVF/ICSI were nulliparous, a status associated with higher risk of adverse obstetric and perinatal outcomes in singleton pregnancies after natural conception, including anemia, preeclampsia, gestational diabetes, placental abruption, preterm birth, interventional delivery, and low birth weight.^5,16^ When nulliparity coincides with AMA, the risk for perinatal complications, such as pregnancy-induced hypertension, placental disorders, and preterm birth, becomes even more pronounced.^7–10,17^

A previous study conducted in Japan revealed that nulliparous women aged 35 years or older, conceiving singleton pregnancies through IVF, exhibited a significantly higher incidence of pregnancy-induced hypertension when compared to their younger counterparts.^18^ This trend was also observed in another recent retrospective study.^19^ In our study, we observed a higher rate of pregnancy-induced hypertension in the SET group in comparison to the DET group. This discrepancy might be attributed, at least in part, to two potential factors: first, the women who received SET were slightly older when they began their IVF/ICSI treatment, and second, most of these women underwent frozen/thawed embryo transfers.

Prior research has indicated an increased risk of pregnancy-induced hypertensive disorders in pregnancies following frozen/thawed embryo transfer.^20,21^ Nevertheless, it is essential to acknowledge that drawing definitive conclusions from this observation remains challenging due to the low number of pregnancy-induced hypertension cases in both groups. Our regression analysis, which encompassed composite pregnancy complications, including pregnancy-induced hypertension, did not identify any significant differences between women of AMA who received either SET or DET, both in crude and adjusted analyses. This observation potentially supports the hypothesis that women in our cohort exhibited favorable health characteristics, including normal antenatal blood pressure, BMI, nonsmoking status, and access to health care, which contribute to overall well-being. These characteristics make them suitable candidates for fertility treatments with a reduced likelihood of experiencing obstetric complications.

A recent meta-analysis has indicated higher cesarean section rates in pregnancies conceived through IVF when compared to naturally conceived pregnancies.^22^ Additionally, a retrospective study conducted in Ireland revealed an increased rate of cesarean section among nulliparous women of AMA when compared to younger women.^23^ Furthermore, a prior investigation demonstrated that nulliparous women of AMA carrying twin pregnancies resulting from IVF had a higher incidence of elective cesarean section compared to those with naturally conceived twins.^14^ However, in our study, we did not identify any significant difference in the mode of delivery between the SET and DET groups, regardless the type of pregnancy. This finding aligns with the results of a previous study, which reported a reduction in cesarean section rates among women of AMA in Sweden and Norway over recent decades. This decline was attributed to improved fetal surveillance techniques.^24^

It is important to note that we chose not to include mode of delivery in our regression analysis due to its complex and multifactorial nature, with many variables influencing this outcome, such as fetal presentation and obstetric indications. Our study primarily focused on composite obstetric and perinatal outcomes, and a detailed analysis of mode of delivery was beyond the scope of this investigation.

While AMA is typically associated with reduced likelihood of achieving pregnancy after IVF/ICSI treatment,^25^ it is noteworthy that the risk of multiple pregnancies remains elevated, particularly when DET is employed in women of this age category.^26^ Multiple pregnancies are known to carry a heightened risk of adverse pregnancy outcomes, especially in women of AMA.^27^ In our study, twin pregnancies were primarily seen in nulliparous women, who more commonly opted for fresh DET, although their overall number was limited. Intriguingly, we found that certain adverse pregnancy outcomes, such as preterm birth and low birthweight, were more significantly prevalent in the DET group, which can be attributed to the increased frequency of twin pregnancies compared to singleton pregnancies. These findings are consistent with prior investigations^27–29^ and underscore the importance of considering the risks associated with multiple pregnancies in women of AMA undergoing DET.

Our study boasts several notable strengths. Foremost among these is the prospective collection of comprehensive data, meticulously recorded within a standardized health care database. This meticulous data collection process not only ensures the accuracy and reliability of the information but also minimizes the potential for recall bias. Furthermore, the study benefits from a standardized maternal care framework that was consistently provided to all women within the study cohort. This approach not only facilitates uniformity in the provision of health care but also enhances the completeness of data capture. As a result, we were able to access and retrieve all pertinent obstetric and perinatal outcome data directly from reliable medical registries, further reinforcing the robustness of our findings.

Additionally, our study's strength is further underscored by the inclusivity of a wide array of treatment and perinatal variables. This comprehensive approach enabled us to explore and analyze various aspects of IVF/ICSI treatment and perinatal outcomes, providing a holistic view of the study population. This breadth of variables not only enhances the depth of our analysis but also permits a nuanced understanding of the factors at play. Consequently, the study's findings are underpinned by a rich and detailed dataset, offering valuable insights into the complex interplay of factors influencing obstetric and perinatal outcomes in women of AMA.

Several limitations should be considered in the interpretation of our study findings. First, the absence of a control group comprising women of AMA who achieved pregnancies through natural conception limits our ability to directly compare outcomes between assisted reproductive technologies and natural conception in this specific age group. Additionally, we lacked information on the mode of previous delivery among multiparous women in our cohort, which could have provided valuable insights into their obstetric history.

Another potential limitation pertains to the absence of data regarding the type of endometrial preparation utilized before frozen/thawed embryo transfer, which could have been a valuable covariate for our regression analyses. Previous research has indicated that pregnancies following hormone replacement therapy for frozen/thawed embryo transfer may be associated with a higher risk of hypertensive disorders of pregnancy when compared to pregnancies following natural cycle frozen/thawed embryo transfer.^30^

Furthermore, it is essential to acknowledge the relatively small sample size of women of AMA included in our study. This limitation arises from the fact that our research was conducted within the framework of a tax-financed health care system at a university hospital-based reproductive medicine center, which set an age limit of 40 years for reimbursed treatment. Consequently, our study could not encompass women of AMA older than 40 years at the time of treatment, potentially excluding a subset of the population with distinct outcomes.

Analyzing composite outcomes in our study offers several advantages, including a comprehensive assessment of complex relationships and increased statistical power when individual outcomes are relatively rare. This approach efficiently captures the overall impact of different variables or interventions, aligning with clinical relevance and practicality. However, it is important to recognize the limitations, such as reduced interpretability, potential heterogeneity, and the loss of information regarding individual outcomes. While composite outcomes enhance the holistic understanding of obstetric and perinatal outcomes in women of AMA, this trade-off should be carefully considered, ensuring alignment with research objectives and clinical context.

## Conclusions

In summary, our study investigated the obstetric and perinatal outcomes of a specific subset of women of AMA aged 39–40 years, without comorbidities who underwent IVF/ICSI treatments with either SET or DET. Although DET was more common in nulliparous women and linked to a higher rate of twin pregnancies, our findings indicate no significant differences in adverse outcomes between the SET and DET groups, even after adjusting for multiple factors. These results underscore the importance of meticulous patient selection and comprehensive maternal care in mitigating potential risks associated with DET in this age subset of women.

Notably, the relatively low number of twin pregnancies in our cohort and the overall good health, without antenatal comorbidities, of the study population may have contributed to the reduced risk of complications. However, we acknowledge the limitations of our study, encompassing the absence of a control group involving natural conceptions and a relatively limited sample size due to age restrictions within the health care system. It is important to note this limitation, acknowledging the challenge of making comprehensive conclusions about women of AMA solely based on this specific age range. Future research should explore these nuances further in larger, multicenter studies to better inform clinical decisions for women pursuing pregnancy at an advanced age.

## Abbreviations Used

AMA: advanced maternal age
AMH: anti-Müllerian hormone
BMI: body mass index
CI: confidence intervals
DET: double embryo transfer
FSH: follicle stimulating hormone
ICSI: intracytoplasmic sperm injection
IQR: interquartile range
IVF: *in vitro* fertilization
OPU: oocyte pick-up
OR: odds ratios
PPROM: preterm prelabor rupture of membranes
SET: single embryo transfer
